# Supplementary material for: A topology-preserving dimensionality reduction method for single-cell RNA-seq data using graph autoencoder
Source: Sci Rep. 2021 Oct 8;11:20028. doi: 10.1038/s41598-021-99003-7 (PMC8501122; doi:10.1038/s41598-021-99003-7)
Supplement: Supplementary file 1 — Supplementary Figures. [file 41598_2021_99003_MOESM1_ESM.pdf]

A topology-preserving dimensionality reduction method for single-cell RNA-seq data using graph autoencoder: supplementary material

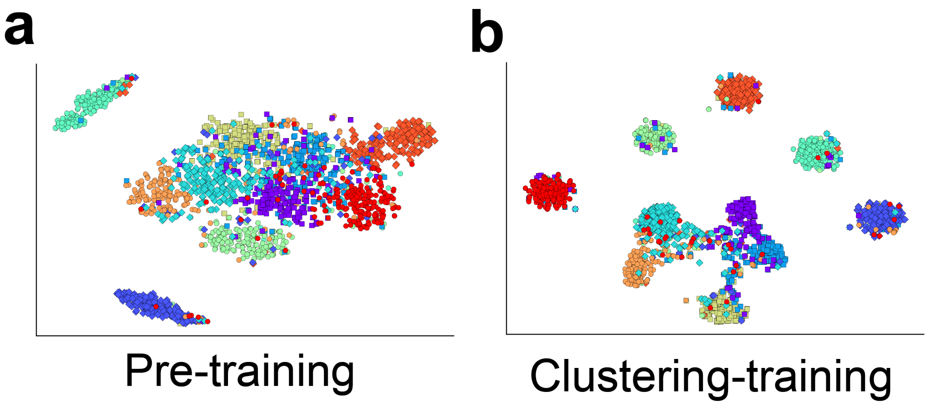

Figure S1: The visualization of data in scenario4 (Fig2d) after pre-training and clustering-training. (a) After pre-training, scGAE cannot separate different clusters clearly. (b) The clustering-training generates a clustering-friendly embedding with compact clusters.

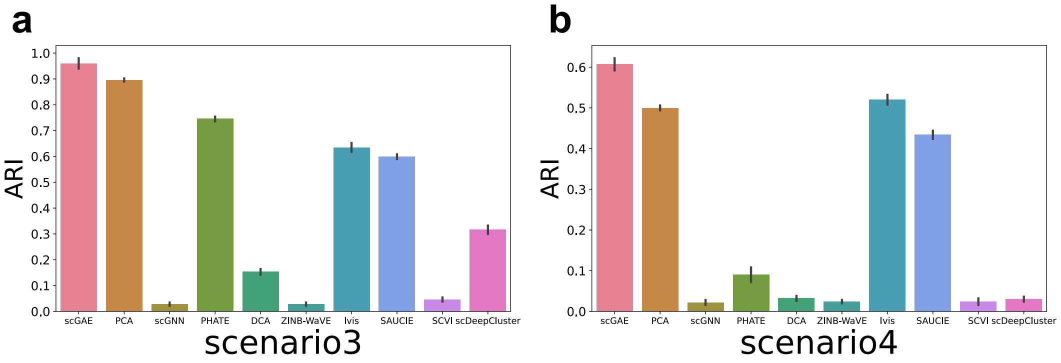

Figure S2: Quantitative evaluation of scGAE and several other competitive methods on clustering tasks. In scenario3 (a) and scenario4 (b), the adjusted rand index (ARI) measures the difference between the ground truth and the inferred clusters.

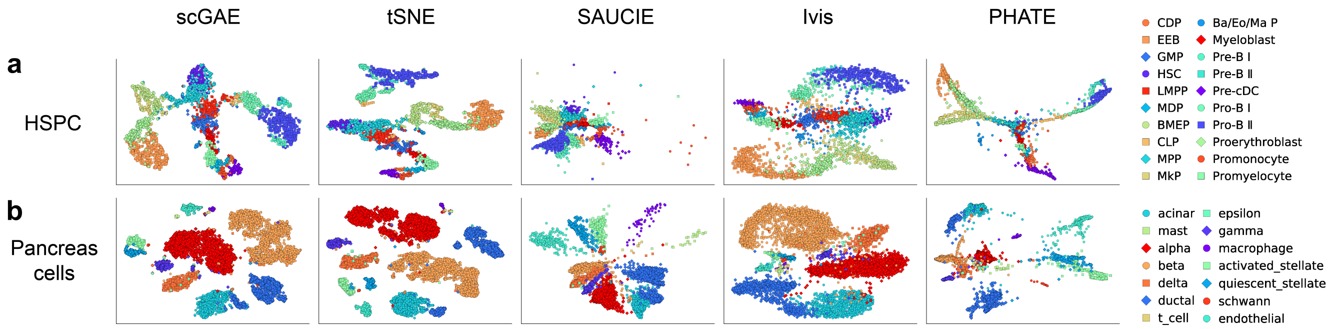

Figure S3: Visualization of the two real datasets by scGAE, tSNE, SAUCIE, Ivis, and PHATE. (a) Visualization of HSPC cells. (b) Visualization of pancreases cells.

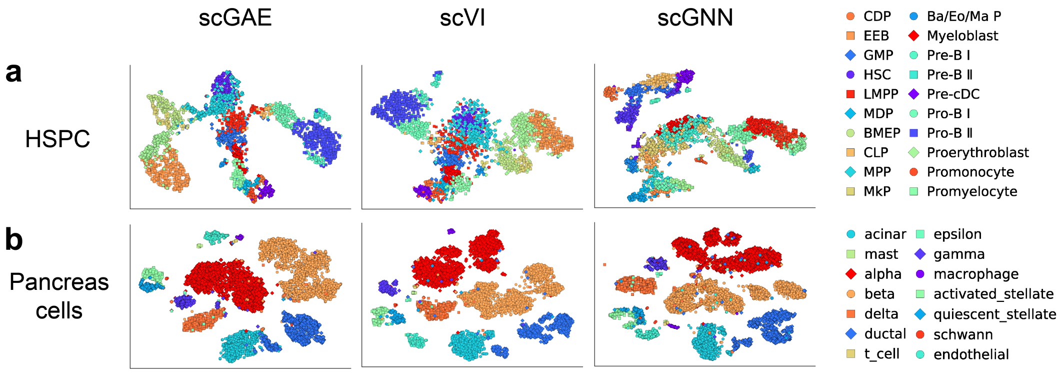

Figure S4: Visualization of the two real datasets by scGAE, scGNN, and scVI. (a) Visualization of HSPC cells. (b) Visualization of pancreases cells.

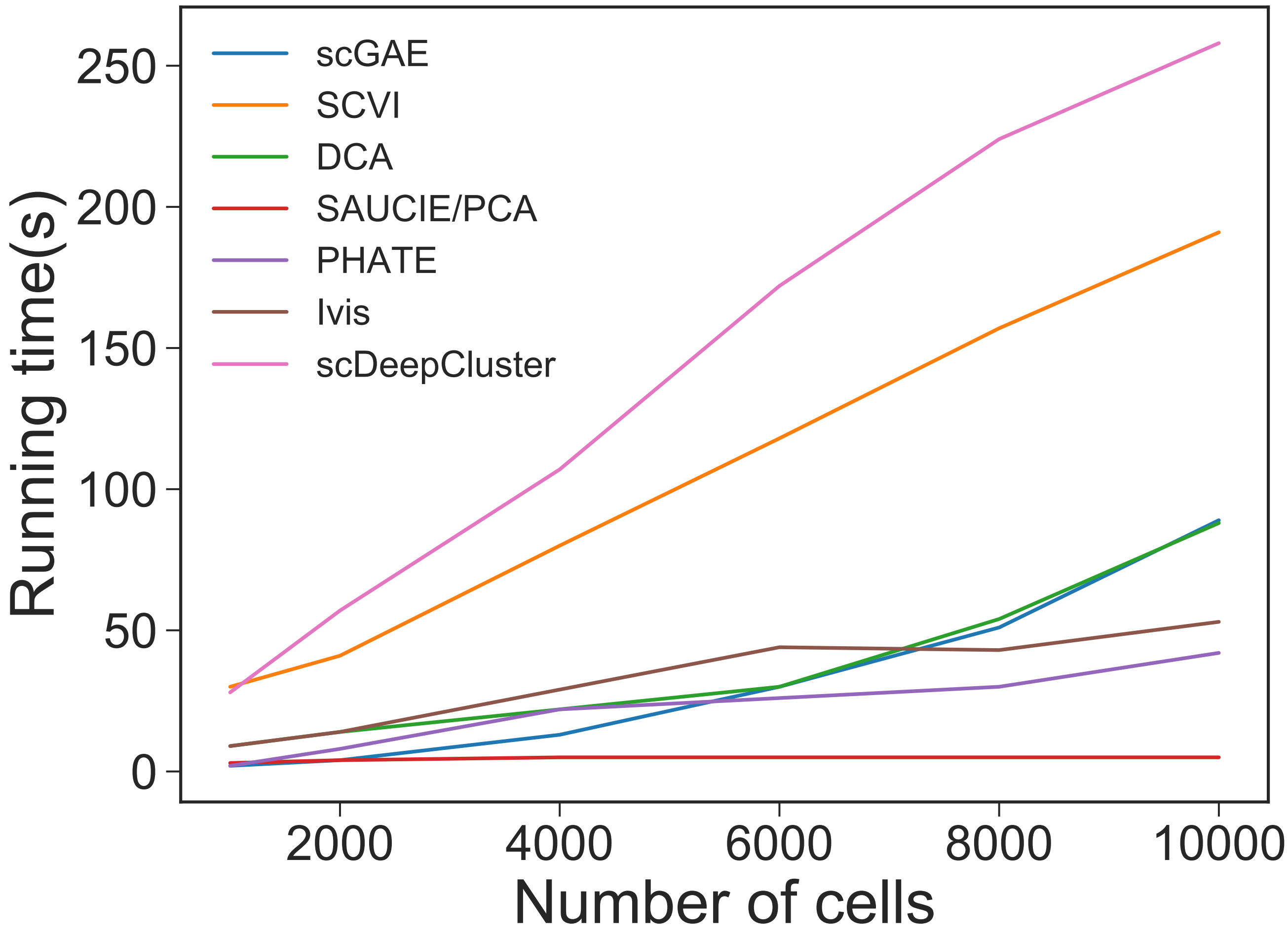

Figure S5: The running time for training scGAE, SCVI, DCA, SAUCIE, PCA, PHATE, Ivis, and scDeepCluster on simulated datasets with 5000 genes and different numbers of cells.

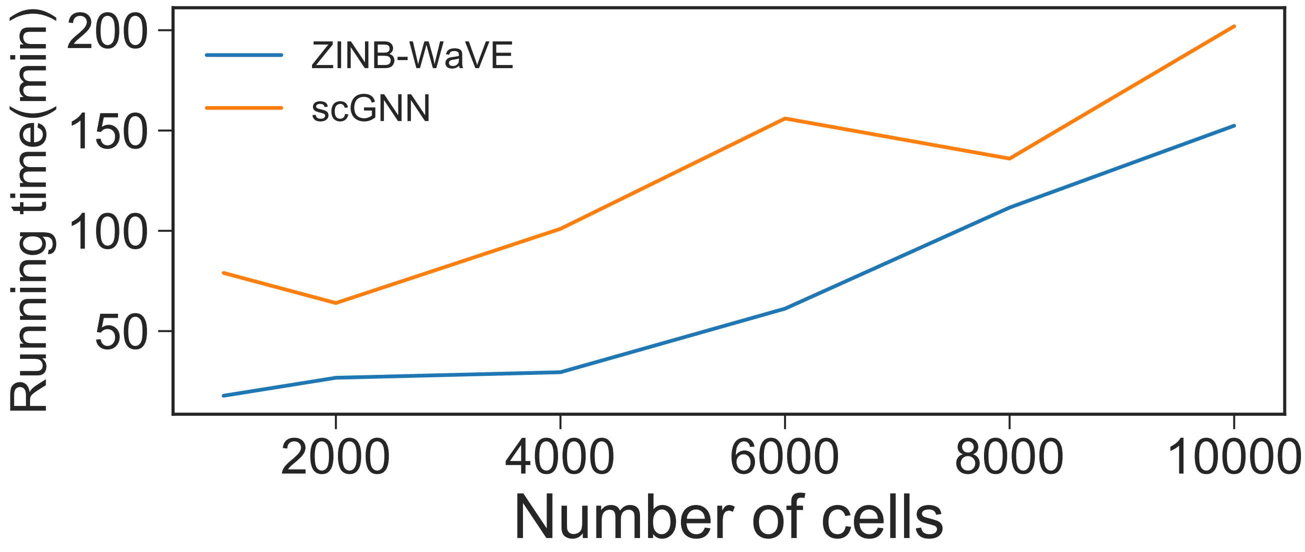

Figure S6: The running time for training ZINB-WaVE and scGNN on simulated datasets with 5000 genes and different numbers of cells.

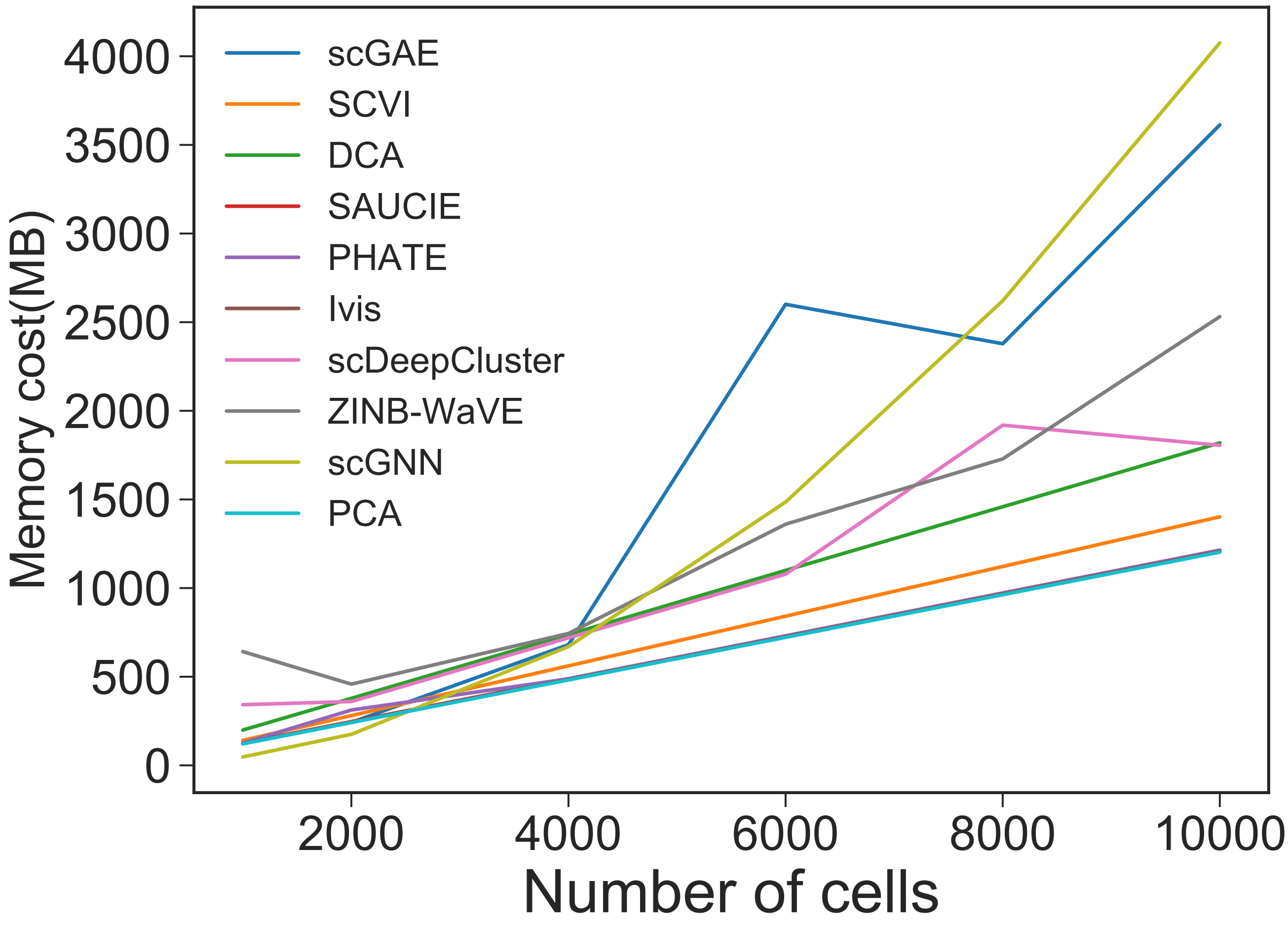

Figure S7: The memory cost for training scGAE, SCVI, DCA, SAUCIE, PCA, PHATE, Ivis, scDeepCluster, ZINB-WaVE and scGNN on simulated datasets with 5000 genes and different numbers of cells.
